# The Role of Mitochondrial Dysfunction and Oxidative Stress in Women’s Reproductive Disorders: Implications for Polycystic Ovary Syndrome and Preeclampsia

**DOI:** 10.3390/ijms26136439

**Published:** 2025-07-04

**Authors:** Evangeline Deer, Babbette LaMarca, Jane F. Reckelhoff, Noha M. Shawky, Kristin Edwards

**Affiliations:** Department of Pharmacology and Toxicology, Women’s Health Research Center, Mississippi Center of Excellence in Perinatal Research, University of Mississippi Medical Center, Jackson, MS 39216, USA; edeer@umc.edu (E.D.); jreckelhoff@umc.edu (J.F.R.); nelsayed@umc.edu (N.M.S.); kedwards1@umc.edu (K.E.)

**Keywords:** pregnancy, polycystic ovary syndrome, preeclampsia, oxidative stress, insulin resistance, endothelial dysfunction, antioxidants, mitochondria

## Abstract

Despite decades of research, the pathophysiology of preeclampsia (PE) and polycystic ovary syndrome (PCOS) remains poorly understood. Notably, no new FDA-approved treatments for PE have emerged in over 50 years. PCOS, a common endocrine disorder, increases a woman’s risk of developing PE. Both conditions share overlapping mechanisms, including insulin resistance, chronic inflammation, endothelial dysfunction, and oxidative stress. While physiological levels of reactive oxygen species (ROS) are essential for reproduction, excess ROS contributes to cellular and mitochondrial damage. This review will assess current evidence linking oxidative stress and mitochondrial dysfunction to the development of PCOS and PE, explore their shared mechanisms, and evaluate emerging therapeutic interventions. Ultimately, a comprehensive understanding of these shared mechanisms may inform strategies for early prediction, prevention, and the treatment of PE and PCOS.

## 1. Introduction

Preeclampsia (PE) is a pregnancy hypertensive disorder that affects 5–7% of pregnancies worldwide and is associated with fetal growth restriction, morbidity, and the mortality of both the mother and fetus [1,2]. Each year approximately 70,000 maternal deaths and 500,000 fetal deaths are linked to preeclamptic pregnancies [2], underscoring the critical need for effective management strategies. Polycystic ovary syndrome (PCOS) affects approximately 10% of reproductive-age women and is characterized by hyperandrogenism, ovulatory dysfunction, and a polycystic ovarian morphology [3]. In addition to reproductive challenges, PCOS is frequently associated with metabolic disturbances such as insulin resistance, obesity, and dyslipidemia. Both PE and PCOS share hallmark features including hypertension, endothelial dysfunction, inflammation, and oxidative stress [4,5,6,7] (Table 1). Additionally, women with PCOS are at an increased risk of developing PE [8], highlighting the interconnected nature of these conditions.

Recently, research efforts have focused on the role mitochondrial oxidative stress in the pathology of various diseases, including PE and PCOS. Oxidative stress is defined as an imbalance between reactive oxygen species (ROS) production and the body’s antioxidant defense mechanisms [9]. While physiological levels of ROS are essential for reproductive processes such as folliculogenesis, oocyte maturation, ovulation, fertilization, and implantation, excessive ROS can lead to cellular damage to lipids, proteins, and DNA, thereby impairing reproductive functions [10,11]. Emerging evidence now places mitochondrial oxidative stress at the center of both PE and PCOS pathogenesis, supported by clinical and preclinical studies [12,13,14,15,16,17,18]. This review uniquely synthesizes these findings to propose that mitochondrial dysfunction serves as a shared mechanistic hub linking metabolic, vascular, and immunologic abnormalities across both conditions. We further present a framework integrating immune-mediated mitochondrial stress, highlighting how PCOS may predispose one to PE via cumulative mitochondrial insults during pregnancy.

## 2. Linking PCOS to PE

PCOS is one of the most common endocrine disorders affecting reproductive-aged women, characterized by hyperandrogenism, ovulatory dysfunction, and a polycystic ovarian morphology [12,13]. PE, a well-known hypertensive disorder of pregnancy, poses significant risks to maternal and fetal health and is a leading cause of morbidity and mortality worldwide [14]. As previously mentioned, women with PCOS are at an increased risk of developing PE during pregnancy due to shared pathophysiological mechanisms, including insulin resistance, hyperandrogenism, chronic inflammation, and endothelial dysfunction [15].

Women with PCOS often exhibit insulin resistance (IR) and hyperinsulinemia, which contribute to systemic inflammation and endothelial dysfunction [16], which are key drivers of PE pathogenesis. Additionally, hyperandrogenism in PCOS alters vascular responsiveness and may exacerbate hypertensive responses during pregnancy, increasing susceptibility to PE [16]. Placental hypoxia in preeclampsia is primarily caused by shallow trophoblast invasion of the maternal endometrium and inadequate remodeling of the spiral arteries, which compromises proper placental perfusion and contributes to disease pathogenesis [17]. Both PCOS and PE are conditions associated with increased oxidative stress, which impairs vascular function and placental development, potentially leading to placental hypoxia, a hallmark of PE. Chronic low-grade inflammation and the elevated levels of pro-inflammatory cytokines that are commonly observed in PCOS further contribute to impaired placental vascular remodeling [18,19], a key feature of PE.

Multiple cohort studies and meta-analyses support the association between PCOS and an increased PE risk. A systematic review and meta-analysis by Kjerulff et al. found that women with PCOS had more than a two-fold increased risk of developing PE compared to women without PCOS, even after adjusting for obesity [20]. This suggests that PCOS-related metabolic and hormonal abnormalities independently contribute to the risk of PE. Moreover, the risk of PE is heightened in PCOS patients who undergo assisted reproductive technologies (ARTs) [21], possibly due to ovarian hyperstimulation and an altered hormonal status. In summary, the interplay of insulin resistance, hyperandrogenism, oxidative stress, and inflammation in PCOS creates a pro-hypertensive state that predisposes affected women to preeclampsia. Therefore, early identification and targeted interventions in PCOS patients, especially during pregnancy, are crucial for mitigating PE risk and improving maternal–fetal outcomes.

## 3. Oxidative Stress and Mitochondrial Function

Oxidative stress is produced from multiple sources of free radicals from NADPH oxidases, nitric oxide synthase, and xanthine oxidase, all contributing to mitochondrial dysfunction [22]. During a normal pregnancy, oxidative stress is important in facilitating placental development [23,24,25]. However, an increase in ROS can lead to abnormal and underdeveloped placental functions, resulting in an inadequate oxygen and nutrient supply to the fetus [24]. This can also promote the adhesion of platelets and leukocytes to the endothelium and the release of antiangiogenic factors and pro-inflammatory cytokines [24]. Given the detrimental effects of oxidative stress, antioxidants have emerged as a potential therapeutic option. Antioxidants have a crucial role in protecting cellular membranes from peroxidation [26], thereby reducing cellular damage and maintaining cellular membrane integrity and suggesting their potential role as a therapeutic option to mitigate cardiovascular disorders.

**Figure 1:**
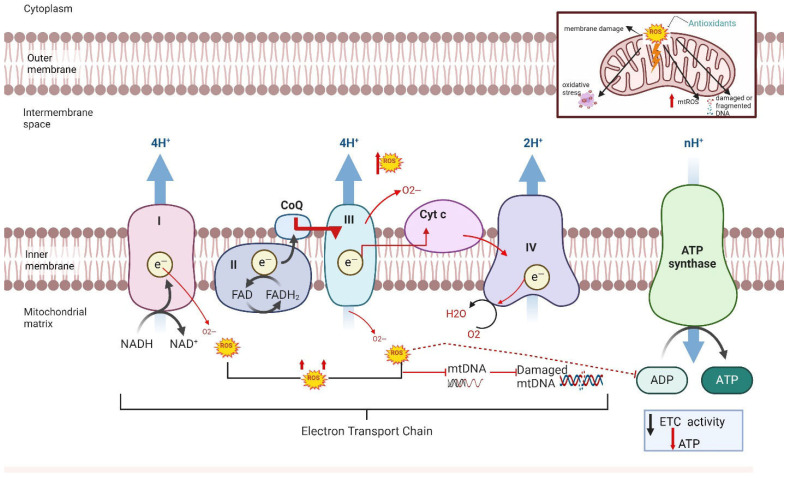
Production of mitochondrial ROS within the electron transport chain. The electron transport chain process includes transferring electrons via redox reactions and coupling with protons (H+) across the mitochondrial membrane generating a proton gradient driving ATP synthase to create adenosine triphosphate (ATP). In preeclampsia (PE), reactive oxygen species are generated by complex I and III resulting in a decrease in electron transport chain (ETC) activity, a loss of membrane potential, and a decrease in ATP production in complex V. These result in membrane damage, the release of cytochrome c, altered signaling, and oxidative damage to mitochondrial DNA and proteins.

Mitochondria are the primary source of intracellular ROS. Under physiological conditions, they produce low levels of ROS that help regulate oxygen sensing, redox homeostasis, and the vascular tone. However, impaired mitochondrial function leads to excessive ROS generation, disrupting electron transport, damaging mitochondrial membranes, and triggering apoptosis [27,28,29,30]. However, when mitochondrial function is impaired, ROS production increases significantly, leading to higher levels than typically seen in physiological conditions [27]. This excess ROS production is caused by impaired electron transport chain (ETC) complexes [29,30,31], which are responsible for oxygen consumption and the production of water in the mitochondria. The accumulation of ROS impairs the electron transport chain’s ability to support efficient respiration, leading to mitochondrial damage (Figure 1). This accumulation of mitochondrial (mt) ROS production can damage the respiratory chain, cause membrane lipid peroxidation, and release of cytochrome c, thereby leading to the initiation of apoptotic pathways and ultimately causing cell death [31,32]. In conditions like PCOS and PE, where oxidative stress and mitochondrial dysfunction are prevalent, this increased ROS production may contribute to organ damage and dysfunction [33]. Our group has contributed to elucidating the role of oxidative stress and mitochondrial dysfunction in the pathophysiology of PCOS [31,33] and PE [34,35,36,37,38]. Building on this foundation, this review advances a novel interpretation that the immune system’s modulation of mitochondrial health through T cell activation, cytokine signaling, and NK cell engagement may act as a central driver of organ-specific dysfunction in both disorders. This perspective offers a new insight into the role of mitochondrial-targeted therapies, not merely as antioxidant strategies, but as modulators of immune–metabolic crosstalk.

### 3.1. Immune Activation, Oxidative Stress, and Mitochondrial Injury in PE

During PE, oxidative stress is associated with a maternal inflammatory response, leading to insufficient placental perfusion and a low birth weight in offspring [39]. The infiltration of immune cells into tissues, such as in the kidney or placenta, causes the release oxidative radicals and can result in tissue damage, ultimately contributing to cell death and culminating in multi-organ dysfunction [40], a key factor in diagnosing PE. As the pregnancy progresses, the maternal endothelium is exposed to placental factors released into the maternal circulation, including vasoconstrictors, oxidative stress molecules, anti-angiogenic factors, and inflammatory mediators, contributing to PE’s multifactorial nature [41]. These factors culminate in renal, cardiovascular, endothelial, and neurovascular dysfunction [42].

An imbalance in the immune response attributed to the increase in CD4+ T helper 1 cells (CD4+ T), pro-inflammatory cytokines, autoantibodies, and oxidative stress occurs in PE [41,42]. Oxidative stress or hypoxic conditions in PE lead to increased levels of trophoblast debris and necrotic trophoblast cells [43]. Regulatory T (Tregs) cells mediate pregnancy tolerance by reducing inflammation and oxidative stress by managing trophoblast engagement and decidual transformation via dendritic cells and natural killer (NK) cells collectively [43,44]. Additionally, pregnancies affected by preeclampsia exhibit a higher percentage of Th17 cells and reduced levels of Treg cells, indicating a shift from Treg to Th17 dominance, accompanied by a parallel shift from Th2 to Th1 immune responses [45]. In women with PE, the imbalance in effector T cells creates a cytotoxic NK cell environment contributing to cellular damage mediated by antibody cytotoxicity leading to cellular mitochondrial dysfunction and apoptosis, which contributes to end-organ damage [46]. Moreover, studies using animal and cell culture models have substantiated the effects of NK-cell-mediated mitochondrial dysfunction and elevated mitochondrial ROS (mtROS) in the placenta, endothelium, and kidney [47,48].

Placental oxidative stress is necessary for a normal pregnancy, regulating energy metabolism, cell proliferation and apoptosis, intracellular and intercellular signaling, and oxidative–reductive processes [49]. The increased maternal and fetal oxygen demand at the mitochondrial level results in the increased generation of free radicals, including the superoxide anion radicals (O_2_^−^), hydrogen peroxide (H_2_O_2_), and hydroxyl anion radicals (OH^−^), produced through mitochondrial oxidative phosphorylation [49] (Figure 1). Moreover, research has demonstrated that in PE, reduced mtROS can damage the placenta [50]. This damage triggers compensatory mechanisms, leading to the increased expression of antioxidant enzymes, such as catalase, glutathione peroxidase, and peroxiredoxin 2, which work to protect against ROS-induced mitochondrial injury [50]. Additionally, at the cellular level, PE has been largely associated with the over-production and release of free radicals by the placenta. Inflammatory cells are responsible for the increased production and release of ROS [51]. Although a balance between ROS and ATP is crucial for homeostasis [28] and successful pregnancy, our models of PE demonstrate that mtROS is causative of hypertension. In early-onset PE patients, we found lower-than-normal levels of placental mtROS and ATP [48], which could contribute to reduced fetal growth, weight, and survival.

Several studies have shown that increased oxidative stress contributes to endothelial dysfunction and lower antioxidant vitamin levels in women with late-onset PE [52,53,54]. Early-onset preeclampsia, which occurs before 34 weeks of gestation, is typically associated with placental dysfunction, impaired trophoblast invasion, and higher rates of fetal complications, whereas late-onset preeclampsia, occurring at or after 34 weeks, is more often linked to maternal cardiovascular or metabolic factors and tends to present with milder clinical features. Antioxidant systems, which can be nonenzymatic (vitamins C and E, uric acid, glutathione, carotene, and flavonoids) or enzymatic (superoxide dismutase, catalase, glutathione oxidase, etc.) can stabilize excess levels of ROS and thus counteract ROS reactivity [54,55] (Figure 1). Antioxidants are a first-line defense mechanism against oxidative stress. Although vitamins C and E were hypothesized as being beneficial for preventing PE [56], supplementation has not been shown to reduce the risk of PE [57,58] and may increase the mother’s risk of gestational hypertension and placental abruption [57]. Vitamin E has been shown to maintain a balance between T helper 1 and T helper 2 cells, which is crucial for promoting the transition from early to late pregnancy [59,60]. However, our clinical studies suggest that antioxidants would not have been beneficial in the prevention of PE due to the early PE placenta having a decrease in mtROS compared to late gestation control placentas [34,48], thus suggesting that antioxidant supplementation may reduce levels to be detrimental for the fetus. Moreover, placentas from women with early-onset PE showed an increase in the mitochondrial unfolded protein response indicating mitochondrial stress [61]. In contrast, supplementation for mitochondrial health and function may be a better possibility to consider for prevention therapy.

### 3.2. The Role of Immune Activation in PE in Contributing to mtROS/ROS

**Figure 2:**
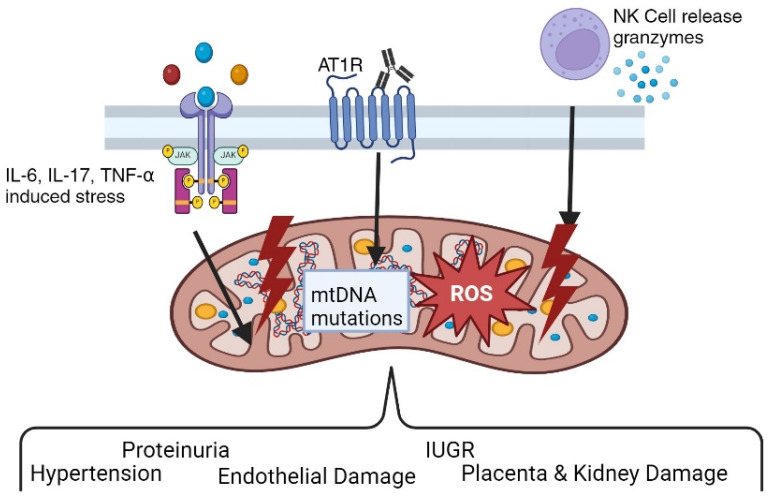
Pathophysiological role of angiotensin II type I receptor autoantibodies (AT1-AAs) in preeclampsia. Various findings by researchers support the hypothesis that preeclampsia is an autoimmune disease characterized by the presence of AT1-AAs. AT1-AAs result in hypertension, oxidative stress, proteinuria, endothelial dysfunction, and other classic features of preeclampsia both in vitro and in vivo.

During early gestation, implantation and placental development rely on glycolysis for ATP production, leading to a low-oxygen environment at the implantation site [62]. As pregnancy progresses, the uteroplacental circulation is established in the second trimester, and oxidative metabolism increases to generate ATP in the placenta in a normal pregnancy [55]. In PE, impaired mitochondrial bioenergetics reduces ATP production and increases mtROS [52]. This oxidative stress contributes to endothelial cell dysfunction, an inadequate oxygen supply, insufficient angiogenesis, and a lack of nitric oxide bioavailability, all hallmark features of PE [63]. Research investigating the role of circulating factors in mtROS and endothelial cell dysfunction has shown that exposing human umbilical vein endothelial cells (HUVECs) to sera from women with preeclampsia (PE) or normal pregnancies reveals significant differences [34,47]. Notably, blocking the agonistic angiotensin II type 1 receptor autoantibody (AT1-AA) can reduce the detrimental effects of PE serum on mitochondrial function [38]. Moreover, the adoptive transfer of CD4+ T helper cells from the reduced uterine perfusion pressure (RUPP) rat model of PE into normal pregnant rats increases circulating inflammatory cytokines, such as TNF-alpha, IL-17, IL-6, and AT1-AA, and mt dysfunction, which was shown to contribute to hypertension by administering two different mitochondrial supplements, MitoQ and MitoTempo [34,35,37] (Figure 2). Blockade with Etanercept [34], the TNF-alpha blocker, and Orencia, a T cell inhibitor, were shown to counteract the outcomes seen with the adoptive transfer of RUPP CD4+ T cells. Additionally, TNF-alpha was found to cause increases in placental and renal mtROS [32] and could stimulate NK cells, which can cause direct damage to cells by damaging mtDNA. Since mtDNA encodes a portion of ETC subunits, any damage to mtDNA could result in further increases in mtROS [37]. In rat models of PE, treatments with supplements that target mitochondrial health were able to reduce mtROS and natural killer cell activation and lowered blood pressure [35].

## 4. Pathophysiological Feedback Loops in PCOS: Mitochondria, Hormones, and ROS

**Figure 3:**
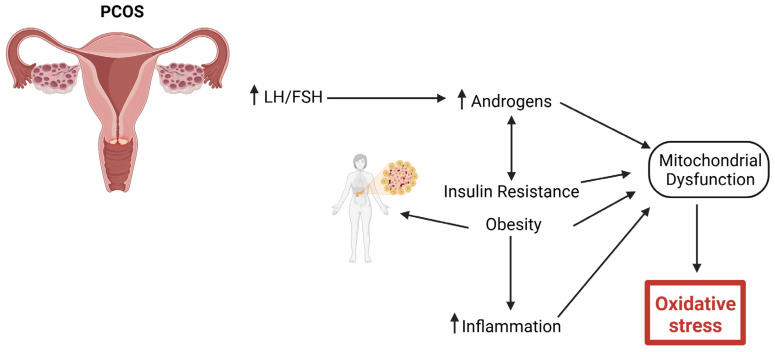
Pathophysiology of PCOS resulting in oxidative stress. Increased hormonal dysregulation, such as increased luteinizing hormone (LH) secretion relative to follicle-stimulating hormone (FSH), results in increased androgens. Insulin resistance (IR) further increases androgens, resulting in obesity, dyslipidemia, and an increased risk of type 2 diabetes mellitus (T2DM). Chronic low-grade inflammation and increased oxidative stress will lead to long-term cardiovascular risks.

As outlined in Figure 3, hormonal dysregulation is central to PCOS. Women with PCOS exhibit the increased secretion of luteinizing hormone (LH) relative to follicle-stimulating hormone (FSH), resulting in elevated androgen levels [13]. Insulin resistance (IR) is another hallmark of PCOS, exacerbating hyperandrogenism and contributing to metabolic disturbances, such as obesity, dyslipidemia, and an increased risk of type 2 diabetes mellitus (T2M) [64,65]. Additionally, PCOS is associated with low-grade chronic inflammation and increased oxidative stress, which contributes to its pathophysiology and long-term cardiovascular risks [66].

The clinical presentation of PCOS is heterogeneous, with symptoms ranging from menstrual irregularities and infertility to acne, hirsutism, and alopecia. The Rotterdam criteria, which require the presence of two out of three features (hyperandrogenism, ovulatory dysfunction, and polycystic ovary morphology) are widely used for diagnosis [67]. Despite its high prevalence and significant health burden, the exact molecular mechanisms underlying PCOS remain incompletely understood, necessitating further research into the syndrome’s complex interplay of metabolic, endocrine, and inflammatory pathways.

**Figure 4:**
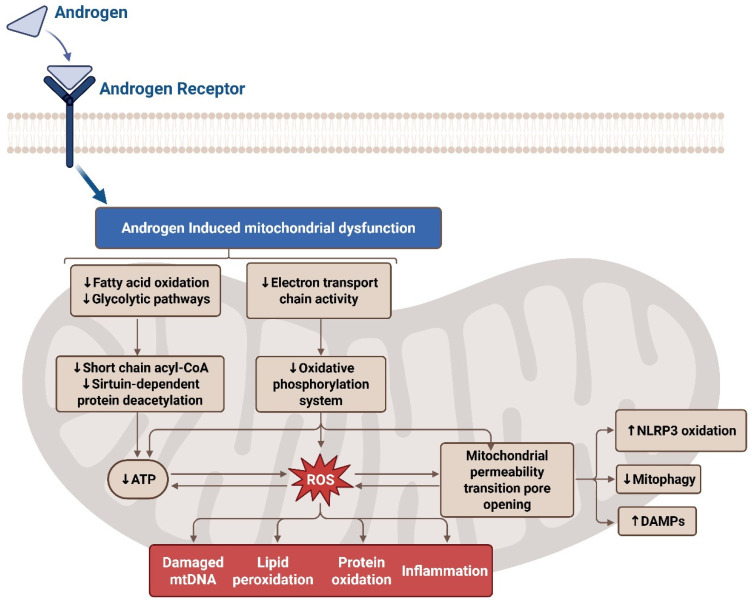
Effects of oxidative stress on the electron transport chain in PCOS. The androgen receptor alters mitochondrial function and acts to decrease glycolysis, ATP, and electron transport chain (ETC) activity by producing mitochondrial reactive oxygen species (mtROS). This leads to damaged mitochondrial DNA (mtDNA), lipid peroxidation, inflammation, and protein oxidation in the mitochondria of PCOS-affected women. Additionally, the mitochondrial permeability transition pore (MPTP) opens leading to increased damage-associated molecular patterns (DAMPs) and NLR family pyrin domain containing 3 (NLRP3) oxidation while decreasing mitophagy.

While mitochondrial dysfunction has been increasingly implicated in PCOS pathophysiology, involving granulosa cell integrity, metabolic substrate handling and inflammatory priming may predispose women with PCOS to adverse pregnancy outcomes. This review is among the first to explore these mitochondrial perturbations with the immunologic landscape of PE, drawing new parallels that may inform integrated therapeutic targets [68]. Recent research highlights altered mitochondrial function in multiple tissues, including the skeletal muscle, adipose tissue, and ovarian cells, contributing to the metabolic and reproductive abnormalities seen in PCOS [28]. In a study by Cree-Green and colleagues, obese girls with PCOS had a distinct metabolic signature during fasting and hyperinsulinemia [68]. Moreover, women with PCOS have a uniquely decreased fasting short-, medium-, and long-chain acylcarnitine, as well as insulin-stimulated valine breakdown products [68]. In PCOS, mitochondria play a crucial role in energy production, steroidogenesis, and the regulation of reactive oxygen species (ROS). Evidence suggests that androgen levels induce changes in metabolism, potentially impairing substrate utilization and energy production [69]. These findings suggest that testosterone may impair mitochondrial substrate handling, contributing to the metabolic abnormalities observed in PCOS (Figure 4).

Mitochondrial (mt) dysfunction in PCOS is characterized by several key alterations, including reduced mitochondrial DNA (mtDNA) content, disrupted mitochondrial biogenesis, impaired oxidative phosphorylation, and excessive ROS production [70]. In granulosa cells, which are crucial for oocyte maturation, mitochondrial abnormalities may impair folliculogenesis and reduce oocyte quality, thus contributing to infertility in PCOS patients [71]. For example, Wang and colleagues observed significantly decreased nicotinamide adenine dinucleotide (NAD+) levels in the granulosa cells of PCOS-affected women, along with increased expression of inflammatory cytokines, increased ROS, decreased ATP generation, and decreased mitochondrial membrane potential [72]. Similarly, Zhang and colleagues reported reduced mitochondrial membrane potential, ATP levels, mtDNA copy numbers, and hypoxia-inducible factor (HIF-1α) mRNA expression in the granulosa cells of PCOS-affected women [73]. Mazloomi and colleagues found elevated ROS levels, a significant decrease in mitochondrial membrane potential, and lower ATP production in granulosa cells [74]. Further studies have highlighted the role of mitochondrial proteins, such as SIRT3, in mitochondrial function. A study by Pang et al. found that the expression of Sirtuin (SIRT3), a soluble protein located in the mitochondrial matrix, was significantly decreased in a PCOS mouse model [75]. This reduction of SIRT3 impairs mt respiration, while increasing both mtROS and insulin resistance [75]. The interaction between insulin resistance and mt dysfunction creates a vicious cycle: defective mitochondria exacerbate insulin resistance, which in turn worsens mitochondrial performance [70]. This bidirectional relationship amplifies oxidative stress and chronic inflammation, further contributing to the metabolic and reproductive issues characteristic of PCOS. Targeting mitochondrial dysfunction through therapeutic strategies such as antioxidants, exercise, and agents that enhance mitochondrial biogenesis may offer potential benefits in improving metabolic and reproductive outcomes in PCOS [76]. Although strong evidence supports mitochondrial involvement in PCOS, most studies are observational, and mechanistic data remain limited. Few studies have addressed how mtDNA mutations or the epigenetic regulation of mitochondrial genes may influence reproductive functions. Further research is needed to validate findings in diverse populations and in longitudinal studies. Several studies have reported that lifestyle interventions, antioxidant supplementation, and insulin-sensitizing drugs like metformin can reduce oxidative stress markers and improve metabolic and reproductive outcomes in PCOS [68,69]. Additionally, mitochondrial-targeted drugs, such as MitoQ and SS-31 (Bendavia), have shown promise in mitigating mitochondrial dysfunction and oxidative stress [77]. Moreover, intervention studies in preeclamptic patients have shown that coenzyme Q10, a key molecule in the electron transport chain, can increase the antioxidative capacity of the placenta and offer a protective effect against preeclampsia development [78]. Consequently, mitochondrial-targeted agents may further enhance metabolic health and reproductive function by directly targeting mitochondrial pathways involved in energy production and antioxidant defense.

**Figure 5:**
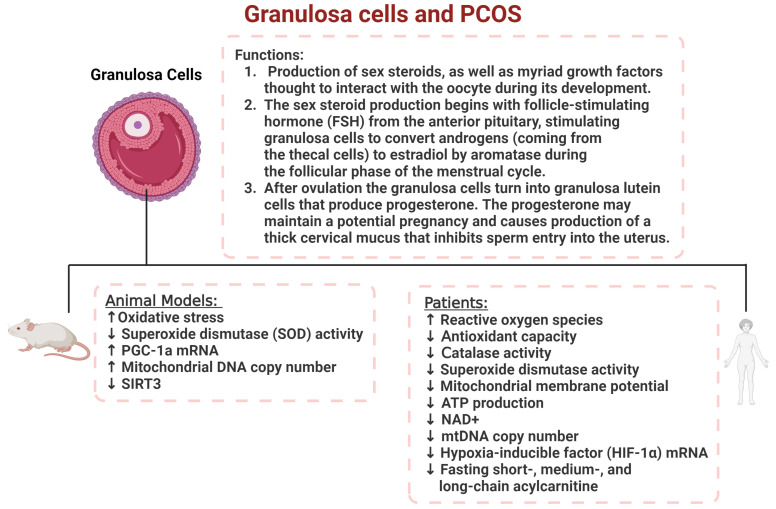
The role of granulosa cells in PCOS women and animal models. Granulosa cell dysfunction contributes to a reduction in steroid hormone production and increased oxidative stress levels resulting in increased ROS activity in both human and animal models of PCOS.

To further explore the mechanism of oxidative stress in PCOS, several rodent models of PCOS and in vitro models that mimic the characteristics of PCOS in women have been developed. A summary of the studies using these models listed below is outlined in Figure 5 Some of these rodent models also exhibit mitochondrial dysfunction and oxidative stress in the ovaries similar to humans. Mansoori and colleagues reported increased oxidative stress, as measured based on thiobarbituric acid assays, and slightly reduced superoxide dismutase (SOD) activity, as measured based on pyrogallol autoxidation, in granulosa cells harvested from female rats treated with letrozole as a model of PCOS [79]. This study also showed that both PGC-1α mRNA and mitochondrial DNA copy numbers were higher in the letrozole-treated rats than in control rats [79]; however, mitochondrial function was not measured. Dehydroepiandrosterone (DHEA) is a widely applied model because it mimics the main features of human PCOS, such as anovulation, alterations in steroidogenesis, and the absence of cyclicity [80]. Li et al. used a DHEA-induced rat model of PCOS to evaluate the role of NADPH oxidase 4 (NOX4), which is highly expressed in ovaries from this model [81] and found that using lentiviral-mediated shRNA against NOX4 reduced oxidative stress in the ovaries, as measured based on 4-hydroxynonenal and MDA, and increased SOD and glutathione peroxidase activities [81]. Presently, this activity has been associated with activation of the Nrf-2/HO-1 signaling pathway [81]. Lai et al. used an in vitro experimental model adding N,N,N′,N′-Tetrakis (2-pyridylmethyl) ethylenediamine (TPEN) to show that zinc deficiency impaired mitochondrial function, leading to increased ROS and acetylated superoxide dismutase 2 (SOD2), which resulted in early apoptosis [82].

Oxidative stress and decreased antioxidant parameters in PCOS are correlated with hypertension, hyperinsulinemia, and dyslipidemia and, therefore, may contribute to metabolic syndrome and cardiovascular disease. A study by Yao et al. found increased superoxide dismutase, glutathione peroxidase 1, catalase, NOX2, NOX4, ROS production, and damaged insulin sensitivity in the skeletal muscles of mice with PCOS [83]. Using a mouse skeletal muscle cell line, C2C12 cells, these authors also found that high levels of testosterone caused mitochondrial dysfunction and increased ROS levels through the androgen receptor (AR)–nicotinamide adenine dinucleotide phosphate oxidase 4 (NOX4) signaling pathway [83]. However, the treatment of C2C12 cells with an antioxidant N-acetylcysteine (NAC) decreased androgen-induced ROS production, improved mitochondrial function, and reversed insulin resistance [83]. The administration of the antioxidant N-acetylcysteine (NAC) to mice with PCOS was also shown to improve insulin sensitivity in the skeletal muscles of the animals [83]. In another study, Yanes, et al. used the dihydrotestosterone (DHT)-treated rat model of PCOS and found that the mRNA expression levels of NOX subunits, p22(phox), p47(phox), gp91(phox), and NOX4 were all upregulated in the kidneys compared to control females [84]; however, mitochondrial function was also not measured in this study.

**Figure 6:**
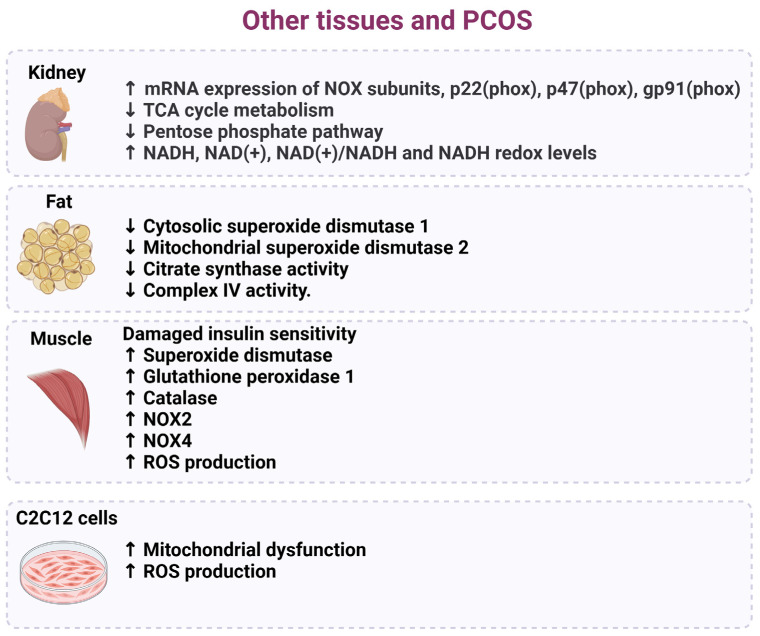
Cellular and tissue dysfunction associated with oxidative stress in PCOS. Several studies have indicated that target tissues and cells in women diagnosed with PCOS exhibit an increase in oxidative stress that harms mitochondrial components, decreases insulin resistance and metabolic pathways, and results in increased ROS production.

Selen et al. performed nuclear magnetic resonance (NMR) metabolomics in the kidneys of a prenatal glucocorticoid-treated mouse model of PCOS as an indication of mitochondrial function [85]. Their results indicated that the TCA cycle metabolism and the pentose phosphate pathway were significantly reduced by 8 to 16 weeks of age in the PCOS model compared with controls. Additionally, they showed that mitochondrial activity was increased, as indicated by the increased levels of NADH, NAD(+), NAD(+)/NADH, and NADH redox [85]. Using the DHT rat model of PCOS, Pruett et al. evaluated the role of oxidative stress in subcutaneous, retroperitoneal, and mesenteric white adipose tissue (WAT) depots and showed that cytosolic SOD 1 and mitochondrial SOD2 proteins were decreased, but catalase was increased in the WAT from the PCOS model compared to controls [33]. They concluded that PCOS and hyperandrogenemia increased oxidative stress and caused the expansion of WAT, which was associated with decreases in mitochondrial function in both subcutaneous and visceral WAT [33]. A summary of the results from this study is outlined in Figure 6. Mitochondria-targeted antioxidants have been shown to modulate the effects of redox signaling and folliculogenesis, thereby attenuating the effects of oxidative stress in a rat model of PCOS [86]. Collectively, this information can be used in the development or discovery of alternative routes of mitochondrial-targeted therapies for PCOS. Cumulatively, the evidence for mitochondrial dysfunction, oxidative stress, and increased inflammation provides a rationale for exploring antioxidant therapies as adjunct treatments, aiming to mitigate systemic inflammation, improve insulin sensitivity, and enhance reproductive function.

## 5. Mitochondrial-Targeted Treatment Options

Mitochondrial dysfunction has a critical role in various reproductive health conditions, including PCOS and PE. As research continues to uncover the importance of mitochondrial health, there is growing interest in developing targeted therapies that specifically address mitochondrial dysfunction. Mitochondrial-targeted treatment options, including antioxidants and innovative therapies, offer potential pathways to mitigate oxidative stress and improve overall health outcomes in affected individuals. Therefore, addressing mitochondrial health through targeted treatments can play a crucial role in improving outcomes for women experiencing PE or PCOS.

Several antioxidants, including vitamins C and E, selenium, and coenzyme Q10, have been investigated for their ability to neutralize ROS and support vascular function. However, large clinical trials, such as the VIP (Vitamin C and Vitamin E in Preeclampsia) trial, found no significant reduction in the incidence of PE with vitamin C and E supplementation and even suggested potential adverse effects [87]. In patients with PCOS, general antioxidant therapies, such as vitamin C, vitamin E, N-acetylcysteine (NAC), and coenzyme Q10 (CoQ10), help mitigate oxidative damage and improve metabolic and reproductive outcomes [86,88,89].

However, given the limitations of conventional antioxidants, targeted mitochondrial therapies have emerged as a promising approach. Mitochondrial-targeted antioxidants such as MitoQ and SS-31 (Bendavia) are designed to selectively accumulate within mitochondria and neutralize ROS at their primary source [90]. MitoQ, a coenzyme Q10 derivative linked to a lipophilic cation, has shown potential in enhancing mitochondrial function, reducing oxidative damage, and improving endothelial health in preclinical studies [91,92]. Similarly, SS-31, a mitochondrial-targeting peptide, has demonstrated protective effects against oxidative-stress-induced mitochondrial dysfunction in hypertensive disorders [92,93]. Among mitochondrial-targeted therapies, agents like mitoquinone (MitoQ), SkQ1, and resveratrol have shown potential in enhancing mitochondrial function by reducing ROS production and improving energy metabolism [94]. These therapies could help restore mitochondrial membrane potential, enhance ATP production, and improve oocyte quality, which are crucial for fertility in PCOS patients [16].

Collectively, these therapies address the underlying oxidative stress and mitochondrial dysfunction in PCOS and PE, offering promising adjunctive strategies to conventional treatments (Table 2). While preclinical findings are encouraging, clinical trials are needed to determine the efficacy, optimal dosing, and timing of these therapies in pregnant women with PE and PCOS. Therefore, studying mitochondrial function in human reproductive tissues is essential, despite the significant methodological challenges it presents. These challenges hinder translational progress in understanding and treating conditions such as polycystic ovary syndrome (PCOS) and preeclampsia (PE). A key issue is the marked heterogeneity among patient populations, including differences in age, the hormonal milieu, comorbidities, and environmental exposures, all of which influence mitochondrial dynamics and oxidative stress responses [95,96]. Moreover, direct access to reproductive tissues such as the placenta or ovarian follicles is often limited—particularly during pregnancy—necessitating the reliance on surrogate biomarkers derived from the peripheral blood or other accessible tissues. However, these biomarkers may not accurately reflect mitochondrial function in the target tissues, leading to the potential misinterpretation of mitochondrial dysfunction [97]. Another underexplored factor is the influence of genetic polymorphisms in mitochondrial DNA (mtDNA), which may modulate mitochondrial bioenergetics, ROS production, and susceptibility to oxidative stress. Recent studies have suggested that mtDNA haplogroups and specific variants could influence individual susceptibility to PE and PCOS, yet such variables are rarely considered in clinical or translational research [98,99]. This omission limits the understanding of interindividual variability in disease presentation and the treatment response, particularly in the context of emerging mitochondrial-targeted therapies such as MitoQ and SS-31. Therefore, future research must integrate mitochondrial genomics and improve access to tissue-specific analyses to enhance the precision and applicability of mitochondrial-based therapeutic strategies in reproductive disorders. Additionally, future research should also explore potential combination therapies that integrate mitochondrial antioxidants with anti-inflammatory, vasoprotective agents, or metabolic treatment options to create a more comprehensive strategy for managing these conditions. Despite promising results in preclinical models, the translation of mitochondrial-targeted antioxidants into clinical therapies remains a challenge. The variability in dosing, timing, and patient selection have limited trial success, highlighting the need for stratified, mechanism-based intervention studies.

## 6. Conclusions and Future Directions

Oxidative stress and mitochondrial dysfunction are now recognized as central drivers in the pathogenesis of both PE and PCOS. In PE, excess ROS contributes to placental hypoxia, endothelial dysfunction, and immune activation, while in PCOS, mitochondrial disturbances impair oocyte quality, hormone regulation, and metabolic homeostasis.

This review contributes a novel perspective by proposing mitochondrial dysfunction as a mechanistic link among the immunologic, endocrine, and vascular features shared by both conditions. We emphasize not only the role of oxidative damage but also the interactions between mitochondrial stress, immune signaling (e.g., Th17/Treg imbalance), and reproductive outcomes.

Despite progress, several research gaps persist (Table 3). The tissue-level assessment of mitochondrial function is limited by ethical and logistical constraints, and mtDNA variants are underexplored despite evidence for their role in disease susceptibility. Additionally, the inconsistent efficacy of antioxidant therapies calls for a shift toward mitochondrial-targeted interventions grounded in a mechanistic understanding.

Future studies should focus on integrating mitochondrial genomics, improving biomarker specificity, and exploring combination therapies that target oxidative stress, inflammation, and metabolic dysfunction simultaneously. Through these avenues, we may unlock more effective strategies to improve reproductive health in women with PCOS and those at risk of PE.

## Figures and Tables

**Table 1:** Shared pathophysiological and clinical characteristics of polycystic ovarian syndrome (PCOS) and preeclampsia (PE).

Risk Factor/Feature	PCOS	PE
Insulin Resistance	Yes	Yes
Obesity	Yes	Yes
Hyperandrogenism	Yes	Potential contributor
Chronic Inflammation	Yes	Yes
Endothelial Dysfunction	Yes	Yes
Oxidative Stress	Yes	Yes

**Table 2:** Summary of mitochondrial-targeted treatments and potential benefits for preeclampsia and PCOS.

Treatment	Description	Clinical Evidence
Antioxidants	Vitamins C and E, selenium, coenzyme Q10 (CoQ10)	Limited efficacy in large clinical trials; general improvement in PCOS outcomes
MitoQ	Coenzyme Q10 derivative with mitochondrial targeting; reduces oxidative damage and enhances mitochondrial function	Preclinical studies show potential in improving endothelial health and mitochondrial function
SS-31 (Bendavia)	Mitochondrial-targeting peptide; protects against oxidative stress-induced mitochondrial dysfunction in hypertensive disorders	Demonstrates protective effects in preclinical settings against oxidative stress
SkQ1	Mitochondrial-targeted antioxidant	Potential in enhancing mitochondrial function by reducing ROS production
Resveratrol	Enhances mitochondrial function; reduces ROS production and improves energy metabolism	Demonstrates promise for improving oocyte quality in patients with PCOS

**Table 3:** Knowledge gaps in the mitochondrial contributions to PE and PCOS.

Area	Knowledge Gap	Research Need
Clinical trials	Few trials on mitochondrial-targeted therapy in pregnant women	Rigorous, stratified trials on safety/efficacy
Genetic variability	Role of mtDNA polymorphisms not routinely studied	Integrate mitochondrial genomics
Biomarkers	Peripheral oxidative markers may not reflect tissue-specific function	Improve tissue-level diagnostic tools

## Data Availability

Data will be made available upon reasonable request.
